# Effects of P-Glycoprotein on the Transport of DL0410, a Potential Multifunctional Anti-Alzheimer Agent

**DOI:** 10.3390/molecules22081246

**Published:** 2017-07-25

**Authors:** Xiaocong Pang, Lin Wang, De Kang, Ying Zhao, Song Wu, Ai-Lin Liu, Guan-Hua Du

**Affiliations:** 1 Institute of Materia Medica, Chinese Academy of Medical Sciences and Peking Union Medical College, Xian Nong Tan Street, Beijing 100050, China; pangxiaocong@imm.ac.cn (X.P.); wlin@imm.ac.cn (L.W.); kangde@imm.ac.cn (D.K.); zhaoyingsky@imm.ac.cn (Y.Z.); ws@imm.ac.cn (S.W.); 2 Beijing Key Laboratory of Drug Target Research and Drug Screening, Chinese Academy of Medical Sciences and Peking Union Medical College, Beijing 100050, China; 3 State Key Laboratory of Bioactive Substance and Function of Natural Medicines, Chinese Academy of Medical Sciences and Peking Union Medical College, Beijing 100050, China

**Keywords:** Alzheimer’s disease, DL0410, P-glycoprotein, homology modelling, molecular docking

## Abstract

In our study, we attempted to investigate the influences of P-glycoprotein (P-gp) on DL0410, a novel synthetic molecule for Alzheimer’s disease (AD) treatment, for intestinal absorption and blood-brain barrier permeability in vitro and related binding mechanisms in silico. Caco-2, MDCK, and MDCK-MDR1 cells were utilized for transport studies, and homology modelling of human P-gp was built for further docking study to uncover the binding mode of DL0410. The results showed that the apparent permeability (Papp) value of DL0410 was approximately 1 × 10^−6^ cm/s, indicating the low permeability of DL0410. With the presence of verapamil, the directional transport of DL0410 disappeared in Caco-2 and MDCK-MDR1 cells, suggesting that DL0410 should be a substrate of P-gp, which was also confirmed by P-gp ATPase assay. In addition, DL0410 could competitively inhibit the transport of Rho123, a P-gp known substrate. According to molecular docking, we also found that DL0410 could bind to the drug binding pocket (DBP), but not the nucleotide binding domain (NBD). In conclusion, DL0410 was a substrate as well as a competitive inhibitor of P-gp, and P-gp had a remarkable impact on the intestine and brain permeability of DL0410, which is of significance for drug research and development.

## 1. Introduction

Alzheimer’s disease (AD), as a progressive neurodegenerative disorder, is the most common cause of memory impairment and dementia in the elderly people [1,2]. Dysfunctions in cholinergic system and other neurotransmitter systems (glutamate and serotonin) were the typical characteristics of AD. Acetylcholinesterase inhibitors (donepezil, galantamine, and rivastigmine) and *N*-methyl-d-aspartate (NMDA) antagonist (memantine) are the currently approved drugs, but all of them do not stop the progression of the disease [3]. Novel therapeutic approaches have emerged over the last years, but most of drugs failed in clinical trials, and cholinesterase inhibitors (ChEI) are still mainstream in the treatment of AD [3,4,5].

In the normal brain, acetylcholinesterase (AChE) occupies 80% of cholinesterase activity, whereas butyrylcholinesterase (BuChE) has a negligible impact on regulating the brain levels of acetylcholine. However, it is inverse in the AD brain, where BuChE activity enhances while AChE activity remains constant or decreases [6]. Therefore, BuChE is also a key enzyme involved in the transmission of nerve signals in the brain. Inhibition of the Histamine 3 receptor (H3R) leads to increasing the release of multiple neurotransmitters, making this receptor an ideal target for the potential enhancement of cognitive processes [7].

DL0410, with a novel scaffold of biphenyl and piperidine, could be a promising multi-target inhibitor for AChE/BuChE and H3R for AD treatment. The patent number of DL0410 was ZL 2007 1 0107604.6. DL0410 could enhance deficits of memory in APP/PS1 transgenic mice, and scopolamine-induced and Aβ1-42-induced amnesia in mice, through inhibiting the activity of cholinesterase, Aβ accumulation, as well as enhancing synapse loss [8,9,10,11,12]. Therefore, DL0410 could be regarded as a candidate drug for AD treatment.

During the development of a new drug, the research of membrane transporters, as a major component of pharmacokinetics, should be emphasized, because membrane transporters have a vital impact on the safety and efficacy profiles of substrate drugs. P-gp, also named as MDR1 and ABCB1, is a transmembrane protein that acts as an ATP-dependent drug efflux pump and causes clinically significant interactions of drugs [13,14]. Drug combination is very common in elderly people, who are also AD sensitive people; in addition, P-gp also has a limitation of substrate drugs that cross into the brain. Therefore, it is necessary to study the P-gp interaction for drugs that target the central nervous system (CNS).

In our study, we reported the synthetic route of DL0410, and investigated the effects of P-glycoprotein on the intestine and blood-brain barrier transport of DL0410 in vitro and the interaction of them in silico for the first time. A human colon carcinoma cell line (Caco-2) was utilized to assess the intestinal absorption of DL0410, and the limitation of permeability caused by P-gp. Madi-Darby Canine Kidney (MDCK) cells and MDCK transfected with the human MDR1 gene (MDCK-MDR1) cells were used to investigate the blood-brain barrier permeability and the interaction of DL0410 with P-gp in vitro. Finally, we built human MDR1 through homology modelling based on Mouse MDR1, and evaluated drug binding affinities by CDOCKER docking tools to uncover the action mechanism between DL0410 and MDR1. This study provides an insight into the mechanisms for the disposition of DL0410 and contributes to avoiding undesired drug-drug interaction (DDI) mediated by P-gp.

## 2. Results

### 2.1. Synthesis of 1,1’-([1,1’-Biphenyl]-4,4’-diyl)bis(3-(piperidin-1-yl)propan-1-one)dihydrochloride (DL0410)

**Figure 1:**
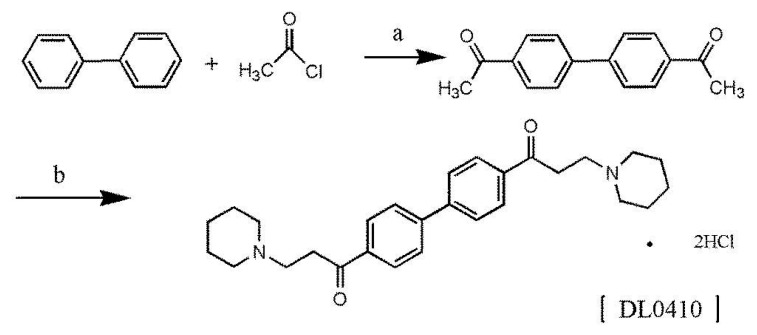
The synthetic route of DL0410. Reagents conditions and yields: (a) CS_2_, AlCl_3_, reflux 4 h, 90%; (b) piperidine hydrochloride, 36–38% HCHO, acetic anhydride, 51.5%.

DL0410 is a novel small symmetric molecule, and we firstly reported the two-step synthetic method here. The chemical structure of DL0410 and synthetic route were outlined in Figure 1. The chemical structure of DL0410 was confirmed by spectroscopy, including ^13^C-NMR (Carbon-13 Nuclear Magnetic Resonance), ^1^H-NMR (Hydrogen-1 Nuclear Magnetic Resonance), IR (Infrared Spectroscopy), MS (Mass Spectrometry), and elemental analysis.

### 2.2. DL0410 Cytotoxicity Profile

**Figure 2:**
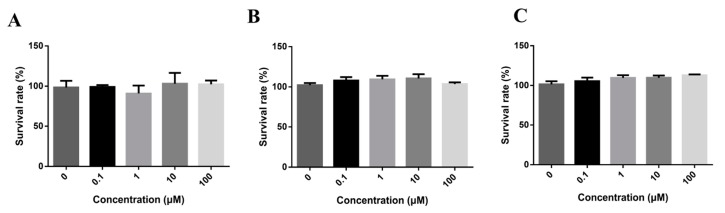
The cytotoxicity of DL0410 on Madi-Darby Canine Kidney (MDCK) cells (**A**) MDCK-MDR1; (**B**) Caco-2; (**C**) DL0410 was tested in a concentration range between 0 and 100 µM for 12 h, using the CCK-8 assay. DL0410 100 µM showed non-toxicity after 12 h. (Mean ± SD, *n* = 3).

In order to identify the doses for transport assays and investigate the cell cytotoxicity of DL0410, we tested a series concentration of DL0410 from 0.1 to 100 µM with Caco-2, MDCK, and MDCK-MDR1, as shown in Figure 2. According to CCK-8 assays, there was no significant cytotoxic effect at 100 µM. 100 µM was chosen for the maximum concentration to continue the further studies.

### 2.3. Transcellular Transport of DL0410 across Caco-2 Cell Monolayer

A Caco-2 cell monolayer, expressing many kinds of transporters, is usually used to assess the intestinal absorption in vitro [15]. Prior to performing the transport studies, we determined the values of Trans Epithelial Electric Resistance (TEER) to evaluate the integrity of the monolayers of the three cell models by EVOM (World Precision Instruments Inc., Sarasota, FL, USA) [16]. Caco-2 cells were seeded for 18 and 21 days, and then the TEER value was approximately up to 1000 Ω·cm^2^. The apparent permeability (Papp) value of fluorescein, as control of hypotonic compound, was 1.089 × 10^−6^ cm/s, and the penetration rate was 0.10%, which satisfied the requirement of further transport assays. DL0410 transport was determined independently across the Caco-2 cell monolayer and compared to the transport as P-gp was inhibited by verapamil, as shown in Table 1. Bidirectional transport was determined to evaluate whether DL0410 transportation was polarized. The value of P_app_ (A→B) increased with the growing concentration of DL0410. But, the P_app_ value of DL0410 was about 1 × 10^−6^ cm/s, implying that intestinal absorption of DL0410 might be not favorable. The influx of DL0410 was greater in the B→A direction than the efflux in the direction of A→B. In addition, the values of the efflux ratio (ER) decrease after the inhibitory effect of verapamil on P-gp.

### 2.4. Transcellular Transport of DL0410 across MDCK, and MDCK-MDR1 Cell Monolayers

MDCK and MDCK-MDR1 cells were further used to study the BBB permeability of DL0410. The cell monolayers were utilized for transport study between days 5 and 7, and the TEER values were up to 240 Ω·cm^2^ or so. The transepithelial transport of fluorescein was utilized as a control to assess the viability of the MDCK and MDCK-MDR1 cell monolayers, and their permeability values (cm/s) were 7.015 × 10^−7^ cm/s and 5.705 × 10^−7^ cm/s, respectively. Then, MDCK and MDCK-MDR1 cells were used to evaluate the BBB permeability of DL0410 and to confirm the effect of P-gp on the permeability of DL0410. In both cell types, DL0410 flux was lower in the A→B direction than in the B→A direction, especially for MDCK-MDR1 cell monolayers. The ER value of DL0410 in MDCK cells was found to be 1.0 approximately, exempting the inhibitory effect of P-gp caused by verapamil (Table 2). However, in MDCK-MDR1 cell, the ER value of DL0410 decreased significantly in the presence of verapamil. In addition, the value ER of DL0410 in MDCK-MDR1 cells was remarkably greater than that in MDCK cells. Net efflux ratio (NER) values of DL0410 were 10.588, 8.770, and 5.556 for the increasing concentration of DL0410 (10, 30, 100 µM), respectively. The saturation of P-gp could contribute to explaining the non-linear relationship between the concentration of DL0410 and ER or NER.

### 2.5. Stimulation of P-gp ATPase by DL0410

**Figure 3:**
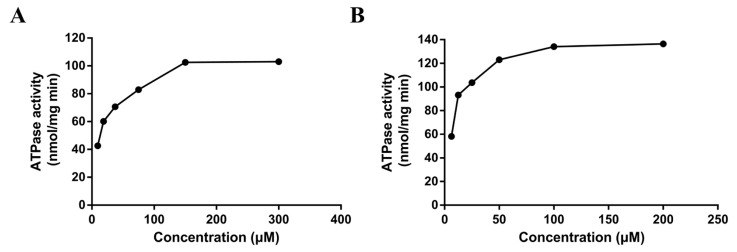
The P-gp ATPase activities on concentration-dependent stimulation by DL0410 (**A**) and verapamil (**B**) in human P-gp membranes. The P-gp ATPase activity was measured through Pi release. The P-gp membranes were exposed to serial concentrations of DL0410 (20–300 µM) and verapamil (2–200 µM). Data are presented as the means of triple determinations.

The ATPase assay is considered a useful tool in vitro to screen P-gp substrates/inhibitors and evaluate the affinity of substrates to P-gp [17]. The rate of ATP consumption stimulated by DL0410 was in a concentration-dependent manner. But, ATP consumption induced by DL0410 was lower than that of verapamil (Figure 3). The Km value of DL0410 and verapamil was 23.53 µM and 10.80 µM, respectively. The Vm value of DL0410 and verapamil was 95.92 µM and 133.20 µM, respectively.

### 2.6. Effects of DL0410 on Rho123 Transport in MDCK-MDR1 Cell

**Figure 4:**
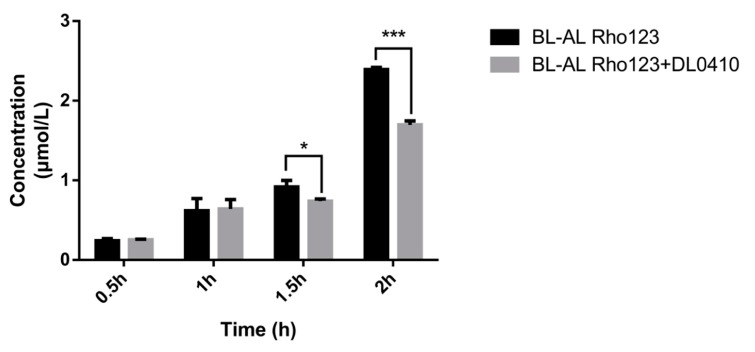
The effects of DL0410 on the transport of Rho123 in MDCK-MDR1 cells. Transport of Rho123 (10 µM) was measured for 2 h in monolayer cultures of MDCK-MDR1 in the absence or presence of DL0410 (50 µM). (Mean ± SD, *n* = 3). The criterion of significance was set at *p* < 0.05 (* *p* < 0.05, *** *p* < 0.001).

Rho 123, a fluorescent P-gp substrate, was used for evaluating the competitive inhibitory effect of DL0410 on P-gp. Rho 123 was added to the basolateral (BL, outside of transwell chamber) side with/without DL0410. The concentration of Rho123 on the apical (AP, inside of transwell chamber) side was determined. The decreasing Rho 123 content on the AP side in the presence of DL0410 was observed at 1.5 h (Figure 4). Subsequently, at 2 h, there was a significant incline of Rho 123 from BL to AL when DL0410 existed on the BL side. Therefore, DL0410 could competitively inhibit P-gp function, and reduced Rho123 efflux.

### 2.7. Homology Modelling of MDR1

**Figure 5:**
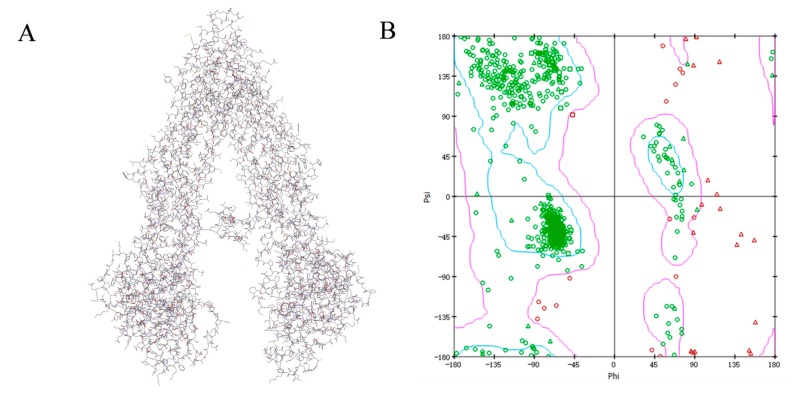
The homology modelled structure (**A**) and Ramachandran map (**B**) of Human MDR1. PDF Total Energy and DOPE Score of P08183.3.M0007 are 7361.354 and −137,194.672, respectively. Profile-3D was used for further evaluation by comparing the identity of the 3D homology model with the amino acid sequence itself. The verify score of P08183.3.M0007 was 411.25. Each point on the Ramachandran Plot represents the φ (phi) and ψ (psi) torsion angles of a residue, indicating that most of amino acid residues were located in the blue and purple zone, which represent the most favorable and favorable regions, respectively. The residues outside the allowed region mainly referred to Gln, Val, and Ser.

The protein sequence of Human MDR1 (Accession ID: P08183) was downloaded from the UNIPROT database (http://www.uniprot.org/downloads). Then, a protein-protein BLAST in NCBI was carried out against the protein data bank (PDB) database to find homologous sequences having resolved 3-dimensional structures and to identify high sequence identity protein structures as template [17]. According to the BLAST search result, we found that mouse MDR1 (PDB ID: 4KSB) was the most similar with human MDR1 with the sequence identity of 87%, therefore 4KSB was used as template for homology modelling. Ten model structures were generated using a modeling module in the Discovery Studio (DS) 2016 package. Based on the PDF total energy and DOPE score, the best model P08183.3.M0007 was selected from the ten models (Figure 5). The final model was validated using Ramachandran Plot and Verify Protein (Profiles-3D). The Ramachandran Plot describes a graphical representation of the local backbone conformation of each residue in a protein. The Ramachandran map suggested that the majority of the amino acid residues were located in the blue area (the most favorable zone) and purple area (the favorable zone), which suggested the reliability of this homology model of human MDR1.

### 2.8. DL0410 Docking to the Drug Binding Pocket of P-gp

**Figure 6:**
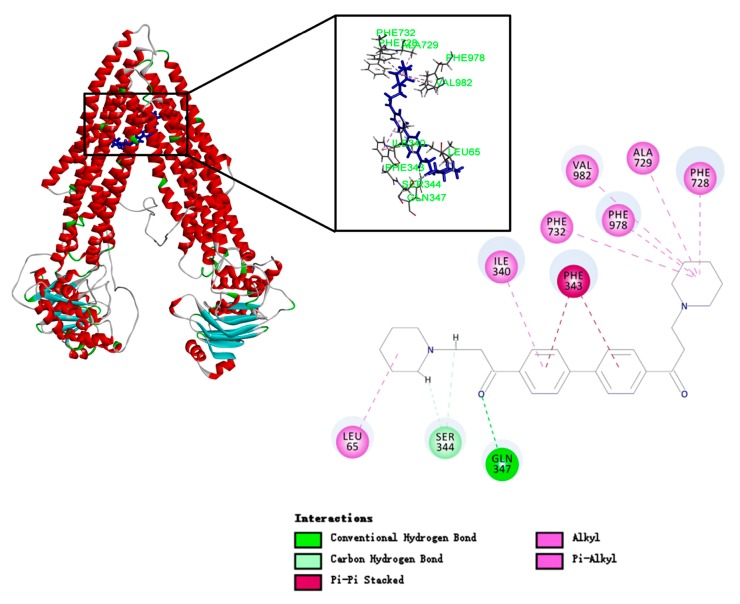
The binding mode of DL0410 to human P-glycoprotein. DL0410 has a good affinity of the drug binding pocket in P-gp. The Figure showed the 3D and 2D interaction. The amino acid residues of the DBP interacting with DL0410 included Leu65, Ile340, Ser344, Phe343, Gln347, Phe728, Ala729, Phe732, Phe978, and Val982 mainly via hydrogen bonding, and π-alkyl and π-π stacked interactions.

**Figure 7:**
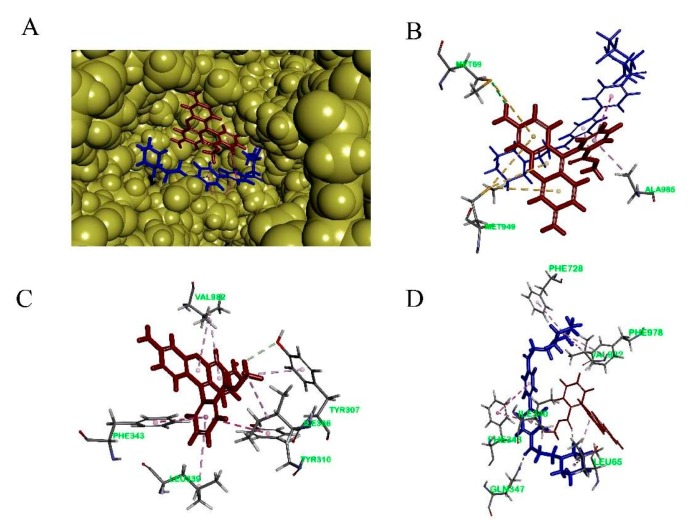
The binding mode of Rho123 to human P-glycoprotein. (**A**): The co-existence of DL0410 and Rho123 on the drug binding pocket of P-gp; (**B**) The 3D interaction model of Rho123 with the drug binding pocket of P-gp in the presence of DL0410; (**C**) The 3D interaction model of Rho123 with the drug binding pocket of P-gp in the absence of DL0410; (**D**) The 3D interaction model of DL0410 with the drug binding pocket of P-gp in the presence of Rho123.

To study the binding mode of DL0410 to P-gp, we utilized the CDOCKER protocol of DS 2016 for docking analysis. As shown in Figure 6 and Table 3, DL0410 had a good affinity of the drug binding pocket (DBP) of P-gp, and interacted with the amino acid residues of Leu65, Ile340, Ser344, Phe343, Gln347, Phe728, Ala729, Phe732, Phe978, and Val982 mainly via hydrogen bonding, π-alkyl, and π-π stacked interaction. But DL0410 failed to bind to the nucleotide binding domain (NBD). Similarly, verapamil, epirubicin, and Rho123 had a preference for the DBP, instead of the NBD, which was consistent with the previous reports [18]. Rho123 could interact with the amino acid residues of DBP, including Ile306, Tyr307, Try310, Phe343, Ile839, and Val982, via π-alkyl and π-π stacked interactions. To have a better understanding of how DL0410 inhibits the efflux of Rho123, we also studied the binding mode of Rho123 in the presence of DL0410 on the DBP of P-gp (Figure 7). With the existence of DL0410, the affinity of Rho123 was reduced and the interactive amino acid residues were changed, mainly referring to Met60, Met949, and Ala985. That may be because DL0410 could block the amino acid residues interacting with Rho123. But DL0410 was not able to dock to the DBP in the presence of verapamil, which further suggests the affinity of DL0410 was lower than that of verapamil.

## 3. Discussion

This work, firstly, gave a description of a new synthesis route of DL0410, which was simple, low-cost, and easily-controlled without high temperature, high pressure, catalytic hydrogenation, or other harsh conditions. Secondly, we mainly investigated the effect of P-gp on the disposition of DL0410, and their interaction. P-gp plays a vital role in the extrusion of drugs, which may lead to poor therapeutic outcomes of drugs in the treatment process [19]. Intestinal absorption is the important component of the pharmacokinetic and toxicokinetic study of drugs utilizing oral administration as the major route of entry into the body. It is also well known that penetrating the BBB is a prerequisite for drugs to cure central nervous system diseases, including AD. Therefore, illustration of the absorption and disposition mechanisms of DL0410 contributes to drug development.

A Caco-2 cell model is usually used for studying transporter-mediated drug intestinal absorption [20]. It was reported that when the value of P_app_ was less than 1 × 10^−6^ cm/s in Caco-2 cells, it indicated a low in vivo absorption (0–20%). A P_app_ value exceeding 10 × 10^−6^ cm/s reveals a high in vivo absorption (70–100%), and P_app_ value between 1 and 10 × 10^−6^ cm/s suggests moderate absorption (20–70%) [15]. Table 1 showed that the P_app_ A→B of DL0410 was approximately 1 × 10^−6^ cm/s, which meant the absorption of DL0410 was not good, which was consistent with our previous in vivo study. Therefore, it suggested that P-gp had an effect on the oral bioavailability of DL0410. The Papp value appeared to be concentration-dependent, whereas the ER value declined with the increase of the concentration, because of the saturation of P-gp.

However, Caco-2 cells express several other multiple transporters besides P-gp, such as the organic anion-transporting polypeptide 2B1 (OATP-B) and the multi-drug resistance-associated protein 2 (MRP2) [21]. The MDCK-MDR1 cell line derived from MDCK cells and transfected with the human MDR1 gene has high P-gp expression and rapid differentiation [22]. Therefore, MDCK and MDCK-MDR1 cells were further used to validate the effects of P-gp on the disposition of DL0410. From Table 2, in the MDCK cell model, we observed that DL0410 could effectively traverse the BBB, but the penetration was not high. Because the expression of P-gp was low in the MDCK cell line, verapamil had no significant effect on the transport of DL0410 through the MDCK cell monolayer. As speculated, the Papp A→B values of DL0410 were remarkably lower than Papp B→A values at all concentrations in MDCK-MDR1 cells, and the phenomenon of receptor saturation existed in the high concentration tested (Table 2). The ER value decreased from 10.323 to 0.298 at 30 µM in the presence of verapamil, suggesting that P-gp engaged in the brain penetration of DL0410. The ER value of Dl0410 in MDCK cells was observed to be lower than that in MDCK-MDR1 cells, which is because of high expression of P-gp in MDCK-MDR1 cells. Therefore, it could be speculated that P-gp participated in the transport of DL0410, affecting intestinal absorption and BBB penetration.

P-gp was an important factor contributing to the occurrence of DDI [23], so it was necessary to evaluate whether the investigational drug was an inhibitor of P-gp. From Figure 3, we observed that Dl0410 could inhibit the transport of Rho123 from the BL side to the AL side in MDCK-MDR1 cells, indicating that it was a substrate as well as an inhibitor of P-gp. These results were consistent with P-gp ATPase activity assay.

When P-gp was stimulated by its substrate, ATP consumption would decrease, reported by luminescence. Verapamil is a stimulatory drug of P-gp ATPase, and known as a P-gp inhibitor because verapamil can inhibit P-gp activity with other substrates by competing for the binding site and their transport. Figure 2 indicated the basal ATPase activity was enhanced by both DL0410 and verapamil, and the activity of ATPase was saturated when the concentration of DL0410 and verapamil reached to 150 µM and 100 µM, respectively. The Km value of verapamil was lower than DL0410, suggesting that the affinity of P-gp for verapamil was stronger than that of DL0410. That was confirmed by the docking results. Like the typical P-gp inhibitors, verapamil and epirubicin, DL0410 could interact with P-gp at the DBP site. However, in terms of the -CDCOKER INTERACTION_ENENGY and -CDCOKER ENERGY, the score of DL0410 was a little lower than that of verapamil and epirubicin, and when verapamil has existed in the DBP already, DL0410 failed to interact with that pocket of P-gp. Because verapamil had a higher affinity with P-gp, it could compete for the amino acid residues in the active site of the DBP and block the interaction between DL0410 and P-gp. DL0410 was also observed to compete with Rho123 for the drug binding site of P-gp, which leads to the decrease of the CDOCKER score. That was consistent with the in vitro result that DL0410 inhibited Rho123 transport in MDCK-MDR1 cells. However, even though Rho123 as a substrate of P-gp exists in the binding pocket, DL0410 still had a good interaction. The presence of Rho123 had no effect on the interaction between DL0410 and P-gp, and the interacting amino acid residues had not significantly changed. Therefore, P-gp could affect the transport of DL0410, and in turn, DL0410 was a competitive inhibitor of P-gp, which should be taken into consideration for avoiding the occurrence of DDI.

## 4. Materials and Methods

### 4.1. Chemicals

DL0410 (purity > 99%) was synthesized by professor Lin Wang and Song Wu in the institute of Materia Medica, Chinese Academy of Medical Sciences. Rho123 and dimethylsulfoxide (DMSO) were purchased from Sigma Chemical Co. (St. Louis, MO, USA). Verapamil and phenacetin (internal standard, IS) was obtained from the National Institutes for Food and Drug Control (Beijing, China). The ATPase Assay Kit was the product of Pgp-GloTM Assay, from Promega (V3601) (Madison, WI, USA). Acetonitrile and methanol were LC-MS grade and obtained from J.T. Baker (Seattle, WA, USA). Formic acid was HPLC-grade, purchased from TEDIA (Fairfield, CA, USA). Ethyl acetate (analytical grade) was obtained from Beijing Chemical Reagent Co. (Beijing, China). Pure water was purchased from Wahaha Company (Hangzhou, China).

### 4.2. Synthesis of DL0410

To a suspension solution of biphenyl (3.08 g, 0.02 M) anhydrous aluminum chloride (2.62 g, 0.02 M) and 20 mL dried carbon disulfide, acetic chloride (1.54 g, 0.02 M) in dried carbon disulfide was added drop by drop with rapidly stirring about 20 min. Stirring was continued for 30 min after addition was complete, and then the reaction mixture was refluxed for 4 h and cooled. The mixture was poured into ice water. The precipitate was filtered, washed with water, and then purified by recrystallization with benzene to obtain 4,4’-diacetylbiphenyl as light-yellow crystals (4.29 g, yield, 90%) mp 188–190 °C (lit [24] 191 °C). A solution of piperidine hydrochloride (1.22 g, 0.01 M), 36–38% HCHO (0.8 g, 0.01 M), and 6 mL acetic anhydride, 4,4’-diacetylbiphenyl (0.6 g, 0.0025 M) were added. The reaction mixture was refluxed for 2 h with stirring and cooled. The reaction solution was concentrated under reduced pressure and the residue as a crude product which was recrystallized twice from a mixture solvent of methanol-ether to give white crystals (0.65 g, yield, 51.5%), m.p. 223–225 °C (dec), elemental analysis C_28_H_36_N_2_O_2_∙2HCl Calc. (%): C 66.53, H 7.58, N 5.54; Found (%): C 65.96, H 7.45, N 5.48. ^13^C-NMR (125 MHz, CDCl_3_):δ 196.3, 143.4, 135.5, 128.8, 127.4, 52.2, 51.1, 33.0, 22.5, 21.3; ^1^H-NMR (400 MHz, DMSO-d_6_): δ(ppm) 10.52 (brs, 2H, D_2_O exchangeable), 8.13 (d, 4H, *J* = 8.4 Hz), 7.98 (d, 4H, *J* = 8.4), 3.72 (t, 4H, *J* = 7.6 Hz), 3.50–3.80 (m, 8H), 2.97–2.86 (m,4H), 1.73–1.69 (m, 10H), 1.40–1.37 (m, 2H). IR (KBr, cm^−1^): 3400, 2940, 2540, 1600, 1605, 1400, 1230, 960. Melting points were determined on a Yanaco micromelting point apparatus and are uncorrected. ^1^H-NMR spectra were recorded on a Varain Mercuy-400 instrument using tetremethylsiline (TMS) as an internal standard (Varian, Salt Lake City, UT, USA). Elemental analyses were performed with a Carlo-Erba 1106 (Carlo-Erba, Milan, Italy). Infrared spectra were recorded with a Nicolet 5700 FT-IR spectrometer with potassium bromide (KBr) pellet (Thermo Electron Corporation, Madison, WI, USA). UPLC-Q-TOF-MS (XEVO G2, Waters Corporation, Milford, MA, USA) identified that the m/z of DL0410 parent ion was 433.2822 (M + H)^+^ and the peak with the highest abundance (*m*/*z* = 217.1573) was affiliated to 1-Phenyl-3-piperidin-1-yl-propan-1-one, which was half of the symmetrical structure of DL0410.

### 4.3. Cell Culture

MDCK and Caco-2 cells were purchased from Cell Center, Institute of Basic Medical Research (Beijing, China). MDCK-MDR1 cell was provided by Professor Shouying Du, School of Materia Medica, Beijing University of Chinese Medicine. Caco-2 was cultured in minimal essential medium (MEM) with 10% fetal bovine serum (FBS), 1% nonessential amino acids, and 100 U/mL antibiotic-antimycotic. MDCK and MDCK-MDR1 cells were cultured in Dulbecco’s modified Eagle’s medium (DMEM) added with 10% FBS and 100 U/mL antibiotic-antimycotic. Cells were cultured in an atmosphere of 5% CO_2_ and 90% relative humidity at 37 °C. Every 3–5 days, cells were digested with 0.5% trypsin and 0.02% EDTA.

### 4.4. Cytotoxicity Study In Vitro

The cytotoxicity of DL0410 was determined by the CCK-8 assay. Caco-2 Cells, at a density of 1 × 10^5^ cells/mL, were seeded in 96-well plates for culture. The seeding density of MDCK and MDCK-MDR1cells were 0.8 × 10^5^ cells/mL. Two days after seeding, increasing concentrations (0.1, 1, 10, 100 µM) of DL0410 were added for treatment, and cytotoxicity was measured after 12 h by the CCK-8 assay.

### 4.5. Transport Assays

Caco-2, MDCK and MDCK-MDR1cells were cultured at seeding densities of 1 × 10^5^, 2 × 10^5^, 2 × 10^5^ cells/cm^2^, respectively, onto polycarbonate membrane transwell inserts in 12-well plates. Prior to performing the transport studies, we determined the values of Trans Epithelial Electric Resistance (TEER) and P_app_ values of fluorescein to ensure the integrity of the monolayers [16]. After seeding for 18 and 21 days, the cultural medium of transwell chambers was removed carefully and washed twice with HBSS (pH 7.4, 37 °C), and then pre-incubated for 30 min at 37 °C. The transwell chambers were incubated for 20 min at 37 °C. To the AP or BL side of the monolayer was added a growing concentration of DL0410 (2.56, 16.67, and 50.00 mM) to initiate the transport experiments. After incubation for 2 h, the liquor of each side was collected for determination. To assess the impact of a P-gp inhibitor on the transport of DL0410, verapamil (50 mM) was added to the AP and BL sides of the monolayer, respectively, before adding DL0410 (16.67 mM). Moreover, the inhibitory effect of DL0410 (100 mM) on P-gp was evaluated by determining the transport of Rho123 (5 mM) from the BL to AP side in the MDCK-MDR1 cell model.

### 4.6. Determination of P-gp ATPase Activity

Pgp-GloTM Assay was performed to evaluate the effect of DL0410 on P-gp ATPase activity. The experiment depends on light-generating reaction by determining the ATP consumption of firefly luciferase [25]. According to the protocol, P-gp-enriched membranes (25 µg) was cultured with DL0410 (9.375, 18.75, 37.5, 75, 150, 300 µM), or verapamil (6.25, 12.5, 25, 50, 100, 200 µM) at 37 °C about 5 min, and then initiate reactions with 5 mM MgATP. Subsequently, the plate was mixed briefly on a plate shaker and incubated for 40 min at 37 °C. After that, the reactions were blocked through initiating luminescence by adding 50 µL of ATP, and then the plate was placed at room temperature for 20 min to allow a luminescent signal to develop. A Spectra Max M5 (Molecular Devices, Sunnyvale, CA, USA) was applied to read the luminescence.

### 4.7. Liquid Chromatography/Mass Spectral Analysis

To analyze DL0410 in HBSS buffer, 15 µL of IS working solution (phenacetin, 1 µg/mL) and 150 µL sample were mixed with 1000 µL ethyl acetate to extract the analytes, and then vortexed for 5 min. After centrifuging at 13,000 rpm for 10 min, the supernatant was added into another tube carefully and evaporated to dryness under a gentle flow of nitrogen gas. The residue was reconstituted in 60 µL acetonitrile–water (20:80, *v*/*v*, containing 0.5% formic acid). After centrifugation, the supernatant was used for analysis. The analytical column was Agilent Zorbax SD-C18 (100 mm × 2.1 mm, 3.5 µm; Agilent Technologies, Santa Clara, CA, USA). The mobile phase was made up of acetonitrile–water (0.5% formic acid) (20:80, *v*/*v*). Injection volume, flow rate, and column temperature were 20 µL, a 0.3 mL/min, and 35 °C, respectively. The mass spectrometer was operated in the positive scan mode. The conditions of ESI source were as follows: drying gas flow was set at 10 L/min, nebulizer pressure was 35.0 psig, capillary voltage was 3000 V, and drying gas temperature was 350 °C. The ESI was performed using nitrogen to assist nebulization. The quantification ions (M + H)^+^ at *m*/*z* 433.28 (140 V) and *m*/*z* 180.22 (90 V) were set for DL0410 and IS, respectively.

### 4.8. Homology Modeling

A protein-protein BLAST was applied against protein data bank (PDB) database to identify homologous sequences with having resolved 3D structures and identify high sequence identical protein structures as template. Then, we obtained the template structure from the RCSB Protein Data Bank (http://www.rcsb.org/pdb/) [26]. Alignment between the sequence of human MDR1 and the template sequence was performed by MODELER in DS 2016 package (Accelrys Software, Inc., San Diego, CA, USA). The best 3D model was selected according to probability density function (PDF) total energy and DOPE score [27]. Lower PDF total energy and DOPE score would indicate a statistically better model. Next, Ramachandran Plot and Verify Protein (Profiles-3D) modules were used for further evaluation.

### 4.9. Molecular Docking

To study the binding mode of DL0410 to P-gp, we utilized the CDOCKER protocol of DS 2016 for docking analysis. The human P-gp homology model was utilized for molecular docking studies and corrected the incomplete residues using the Prepare Protein tool of DS, and then the protein was refined with CHARMm. The structures of verapamil, epirubicin, and Rho123 were downloaded from the NCBI PubChem database and prepared by DS 2016 through adding hydrogen, conversing into 3D structures, PH based ionization and charge neutralization. There were two active pockets of P-gp, including drug binding pocket (DBP) and nucleotide binding domain (NBD). Amino acid residues of the DBP included His61, Gly64, Leu65, et al. The NBD binding site residues were identified to be Typ1044, Val1052, Gly1070, et al. [18].

### 4.10. Statistical Analysis

The Papp values (cm/s) were computed according to the following equation [16],

$$P_{app}=\frac{V}{C_{0}}\times \frac{1}{S}\times \frac{dC}{dt}$$

(*V*: volume of medium in the receiver chamber (mL); *C*_0_: initial concentration in the donor chamber (µM/mL), *S*: the surface area of monolayer (cm^2^); *dC/dt*: variance rate in the receptor chamber of concentration with time (µM/mL/s)) The *ER* was calculated by the *P_app_* value from BL to AP divided by *P_app_* value from AP to BL. In MDCK-MDR1 and MDCK cell models, the *NER* was measured by comparing the *ER* values as follows [16].

(1) $$ER=\frac{P_{app(B-A)}}{P_{app(A-B)}}$$

(2) $$NER=\frac{ER_{MDCK-MDR1}}{ER_{MDCK}}$$

All data were expressed as the mean ± SD. The statistical significance of differences in the transport of Rho123 in the presence of DL0410 was analyzed by paired-samples *T*-tests. GraphPad Prism 6 (GraphPad Software, San Diego, CA, USA) was applied for all analyses.

## 5. Conclusions

In summary, we reported the novel two-step synthesis route of DL0410 for the first time and investigated the interaction between DL0410 and P-gp. P-gp was capable of limiting the intestinal absorption and brain penetration of DL0410, and DL0410 was a substrate as well as a competitive inhibitor of P-gp, which could lead to competing with other P-gp-related substrates. Therefore, it was noteworthy that DDIs should be considered for the further efficacy and safety studies of DL0410.

## Figures and Tables

**Table 1:** The apparent permeability for DL0410 (10–100 µM) in Caco-2 cell monolayer. (Mean ± SD, *n* = 3).

Conditions	P_app_A→B (10^−6^ cm/s)	P_app_B→A (10^−6^ cm/s)	ER
100 μM	1.280 ± 0.017	2.384 ± 0.138	1.858
30 μM	0.841 ± 0.098	3.450 ± 0.619	4.103
10 μM	0.725 ± 0.184	3.275 ± 0.251	4.518
30 μM + verapamil (50 μM)	1.464 ± 0.201	1.949 ± 0.271	1.331

**Table 2:** The transcellular transport of DL0410 across MDCK and MDCK-MDR1 cell monolayers. (Mean ± SD, *n* = 3).

Conditions	MDCK			MDCK-MDR1			NER
	P_app_ (10^−6^ cm/s)		ER	P_app_ (10^−6^ cm/s)		ER	
	A→B	B→A		A→B	B→A		
100 μM	0.950 ± 0.116	0.924 ± 0.005	0.973	0.321 ± 0.039	1.733 ± 0.105	5.406	5.556
30 μM	0.740 ± 0.023	0.871 ± 0.068	1.177	0.310 ± 0.021	3.205 ± 0.061	10.323	8.770
10 μM	0.502 ± 0.103	0.708 ± 0.114	1.410	0.297 ± 0.111	4.434 ± 0.376	14.929	10.588
30 μM + verapamil (50 μM)	0.794 ± 0.057	0.761 ± 0.022	0.958	1.055 ± 0.0414	0.314 ± 0.056	0.298	0.311

**Table 3:** The CDOCKER analysis of verapamil, epirubicin, Rho123, and DL0410 at the DBP site of P-gp.

Compound	-CDCOKER ENERGY	-CDCOKER INTERACTION_ENENGY
Verapamil	7.396	37.663
Epirubicin	13.898	39.036
Rho123	26.988	30.668
Dl0410	5.500	36.275
Rho123(in the presence of DL0410)	11.324	24.717
Dl0410(in the presence of Rho123)	12.830	45.560
Dl0410(in the presence of verapamil)	-	-
